# Dr. David Roberts

**Published:** 1891-11

**Authors:** 


### DR. DAVID ROBERTS.

Dr. David Roberts died suddenly on September 30, 1891, having been actively engaged at his desk until eleven o'clock the previous evening.

Dr. Roberts was a native of Delaware, having been born in the old town of New Castle, November 17, 1822. His father, Joseph Roberts, filled the office of Prothonotary of the Court of Common Pleas of that county. The son was a self-made man, having received an ordinary English education in a school in New Castle. He left home at fifteen, and after pursuing various avocations commenced the study of dentistry with Dr. Gilliams, of Philadelphia. He began practice in 1849, on Spruce Street, near Fourth.

On the establishment of the Philadelphia College of Dental Surgery, in 1852, he entered as a student, and graduated in 1854. (This college must not be confounded with the present one bearing that name. The legitimate successor is the Pennsylvania College of Dental Surgery.)

The subject of this sketch was very successful as a practitioner, and by careful business habits and judicious investments early acquired a competency, and retired from practice in 1878.

His interest in his profession continued, being manifested in many ways. In a recent conversation with the writer, he expressed a fear lest he might be considered as one outside of dentistry, to which he had devoted his best years.

For a long period, and up to the close of his life, he acted as secretary of the board of trustees of the Pennsylvania College of Dental Surgery, and was otherwise actively engaged in furthering the interests of the college and dental education in general.

Dr. Roberts's genial character, together with his marked personality, will cause a vacancy in dental circles in Philadelphia, already heavily afflicted by the loss of a number of its prominent workers.

Dr. Roberts married Miss South, of Philadelphia, in 1858, who survives him. There are no children. Five brothers and a sister are living,—viz., Rev. Edward Roberts, Alfred, James B., General Joseph Roberts, U.S.A., of Philadelphia, Henry Roberts, of Washington, and Miss Margaret Roberts, of Philadelphia.

Dr. Roberts's funeral took place at his residence, 214 West Logan Square, October 3; interment at Laurel Hill Cemetery.

